# 4,4′-Bipyridine–cyclo­hexane-1,2,4,5-tetra­carb­oxy­lic acid (1/1)

**DOI:** 10.1107/S1600536810039024

**Published:** 2010-10-09

**Authors:** Jian-Qiang Liu

**Affiliations:** a Guangdong Medical College, School of Pharmacy, Dongguan 523808, People’s Republic of China

## Abstract

In the title 1:1 adduct, C_10_H_8_N_2_·C_10_H_12_O_8_, the dihedral angle between the pyridine rings in the 4,4-bipyridine molecule is 8.33 (13)°. In the crystal, the cyclo­hexane-1,2,4,5-tetra­carb­oxy­lic acid mol­ecules inter­act with each other through inter­molecular O—H⋯O hydrogen bonds, forming an infinite chain along the *a* axis, which is further linked perpendicularly by O—H⋯N hydrogen bonds involving bipyridine, resulting in a supra­molecular corrugated sheet parallel to the (110) plane.

## Related literature

For background to crystal engineering, see: Desiraju (1989 ▶); Schultheiss *et al.* (2010 ▶); Ebenezer & Muthiah (2010 ▶); An *et al.* (2010 ▶). For a related flexible tetracarboxylic acid, see Holmes *et al.* (1987 ▶); Wang *et al.* (2009 ▶). For a related structure, see: Bhogala *et al.*(2005 ▶).
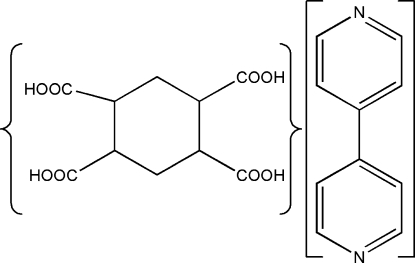


## Experimental

### 

#### Crystal data

C_10_H_8_N_2_·C_10_H_12_O_8_
*M* *_r_* = 416.38
Monoclinic, 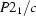

*a* = 12.345 (3) Å
*b* = 9.724 (2) Å
*c* = 16.497 (4) Å
β = 106.364 (3)°
*V* = 1900.1 (8) Å^3^
*Z* = 4
Mo *K*α radiation
μ = 0.11 mm^−1^
*T* = 298 K
0.22 × 0.15 × 0.08 mm

#### Data collection

Bruker APEXII area-detector diffractometer
Absorption correction: multi-scan (*SADABS*; Sheldrick, 2008 ▶) *T*_min_ = 0.975, *T*_max_ = 0.991
9330 measured reflections
3416 independent reflections
2243 reflections with *I* > 2σ(*I*)
*R*_int_ = 0.032

#### Refinement

*R*[*F* ^2^ > 2σ(*F* ^2^)] = 0.047
*wR*(*F* ^2^) = 0.143
*S* = 1.05
3416 reflections
275 parameters
H-atom parameters constrained
Δρ_max_ = 0.26 e Å^−3^
Δρ_min_ = −0.22 e Å^−3^

### 

Data collection: *APEX2* (Bruker, 2008 ▶); cell refinement: *APEX2*; data reduction: *APEX2*; program(s) used to solve structure: *SHELXS97* (Sheldrick, 2008 ▶); program(s) used to refine structure: *SHELXL97* (Sheldrick, 2008 ▶); molecular graphics: *ORTEPIII* (Burnett & Johnson, 1996 ▶), *ORTEP-3 for Windows* (Farrugia, 1997 ▶) and *PLATON* (Spek, 2009 ▶); software used to prepare material for publication: *SHELXL97*.

## Supplementary Material

Crystal structure: contains datablocks I, global. DOI: 10.1107/S1600536810039024/dn2606sup1.cif

Structure factors: contains datablocks I. DOI: 10.1107/S1600536810039024/dn2606Isup2.hkl

Additional supplementary materials:  crystallographic information; 3D view; checkCIF report

## Figures and Tables

**Table 1:** Hydrogen-bond geometry (Å, °)

*D*—H⋯*A*	*D*—H	H⋯*A*	*D*⋯*A*	*D*—H⋯*A*
O1—H1⋯N1^i^	0.82	1.81	2.630 (3)	177
O4—H4⋯N2	0.82	1.86	2.678 (3)	175
O5—H5⋯O2^ii^	0.82	1.96	2.723 (2)	153
O8—H8⋯O7^iii^	0.82	1.82	2.641 (2)	174

Symmetry codes: (i) 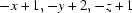; (ii) 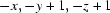; (iii) 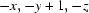.

**Fig. 1.**
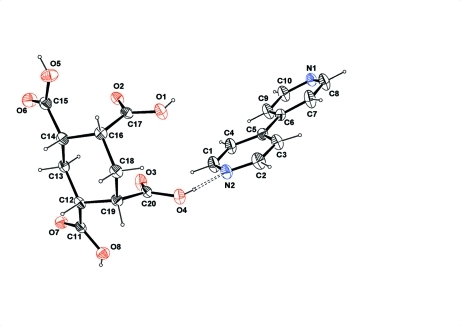
Molecular structure of (I), showing the atom-labelling scheme. Ellipsoids are drawn at the 30% probability level. H atoms are represented as small spheres of arbitrary radii. Hydrogen bond is shown as dashed line.

**Fig. 2.**
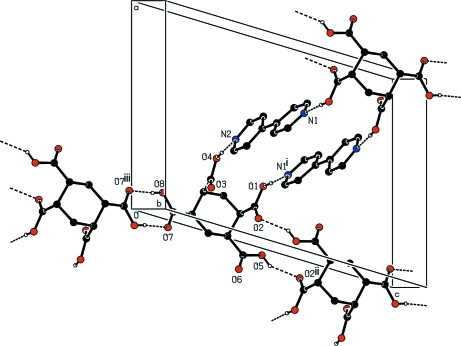
Partial packing view showing the formation of the sheet through O—H···O and N—H···O hydrogen bonds displayed as dashed line. H atoms not involved in hydrogen bonding have been omitted for clarity. [Symmetry codes: (i) -x + 1, -y + 2, -z + 1; (ii) -x, -y + 1, -z + 1; (iii) -x, -y + 1, -z.]

## supplementary crystallographic information

### Comment

The study of non-covalent interactions, such as hydrogen bonding, plays an important role in molecular assembly and crystal engineering (Desiraju, 1989; Schultheiss *et al.*, 2010; Ebenezer & Muthiah, 2010). The simplest cyclohexane-carboxylic acid was firstly employed in the area of coordination chemistry, and many metal–organic frameworks containing cyclohexane-polycarboxylate ligands have been obtained (Holmes *et al.*, 1987; Wang *et al.*, 2009). Furthermore, the cyclohexane-1,2,4,5-tetracarboxylic acid (H_4_L) with H-bond donor/acceptor groups provides inter- and intramolecular H-bonding interactions with N-donor ligands, a driving force for the assembly of polymeric motifs (An *et al.*, 2010). Initially, we attempted to use H_4_L and 4,4'-bipyridine as co-ligands in the presence of Cu^II^ ion, unfortunately, we only obtained the title compound.

The asymmetric unit contains two molecules the 4,4'-bipyridine and the cyclohexane-1,2,4,5-tetracarboxylic acid (H_4_L) connected through O—H···N hydrogen bond (Fig. 1). The cyclohexane-1,2,4,5-tetracarboxylic acid molecule interacts with symmetry related molecules through intermolecular O—H···O hydrogen bonds (Table 1), forming a chain parallel to the a axis. These chains are further linked by O—H···N hydrogen bonds involving the bipyridine resulting in a supramolecular corrugated sheet parallel to the (110) plane (Fig. 2, Table 1). Distances and angles agree with related compounds (Bhogala *et al.*, 2005). It is interesting to note that the cyclohexane-1,2,4,5-tetracarboxylic acid is chiral with four stereogenic center corresponding to the RSRS/SRSR diastereoisomer.

### Experimental

A mixture of Cu(AC)_2_.H_2_O (23 mg, 0.1 mmol), H_4_L (24 mg, 0.1 mmol), 4,4'-pyridine (16 mg, 0.1 mmol), NaOH (0.1 mmol) and 10ml H_2_O was stirred for 2 h, and then the mixture was transferred to a 25 ml Teflon-lined reactor and kept under autogenous pressure at 423 K for 5 d. After the reactor was slowly cooled to room temperature over, the title compound was obtained.

### Refinement

All H atoms attached to C and O atoms were fixed geometrically and treated as riding with C—H = 0.98 Å (methine), 0.97 Å (methylene) or 0.93 Å (aromatic) and O—H = 0.82 Å with U_iso_(H) = 1.2U_eq_(C) or U_iso_(H) = 1.5 U_eq_(O) .

### Crystal data

**Table:**

C_10_H_8_N_2_·C_10_H_12_O_8_	*F*(000) = 872
*M**_r_* = 416.38	*D*_x_ = 1.456 Mg m^−^^3^
Monoclinic, *P*2_1_/*c*	Mo *K*α radiation, λ = 0.71073 Å
Hall symbol: -P 2ybc	Cell parameters from 3417 reflections
*a* = 12.345 (3) Å	θ = 1.7–25.2°
*b* = 9.724 (2) Å	µ = 0.11 mm^−^^1^
*c* = 16.497 (4) Å	*T* = 298 K
β = 106.364 (3)°	Block, colourless
*V* = 1900.1 (8) Å^3^	0.22 × 0.15 × 0.08 mm
*Z* = 4	

### Data collection

**Table:**

Bruker APEXII area-detector diffractometer	3416 independent reflections
Radiation source: fine-focus sealed tube	2243 reflections with *I* > 2σ(*I*)
graphite	*R*_int_ = 0.032
φ and ω scan	θ_max_ = 25.2°, θ_min_ = 1.7°
Absorption correction: multi-scan (*SADABS*; Sheldrick, 2008)	*h* = −14→14
*T*_min_ = 0.975, *T*_max_ = 0.991	*k* = −11→11
9330 measured reflections	*l* = −19→14

### Refinement

**Table:**

Refinement on *F*^2^	Primary atom site location: structure-invariant direct methods
Least-squares matrix: full	Secondary atom site location: difference Fourier map
*R*[*F*^2^ > 2σ(*F*^2^)] = 0.047	Hydrogen site location: inferred from neighbouring sites
*wR*(*F*^2^) = 0.143	H-atom parameters constrained
*S* = 1.05	*w* = 1/[σ^2^(*F*_o_^2^) + (0.0639*P*)^2^ + 0.4389*P*] where *P* = (*F*_o_^2^ + 2*F*_c_^2^)/3
3416 reflections	(Δ/σ)_max_ < 0.001
275 parameters	Δρ_max_ = 0.26 e Å^−^^3^
0 restraints	Δρ_min_ = −0.22 e Å^−^^3^

### Special details

**Table:**

Geometry. All esds (except the esd in the dihedral angle between two l.s. planes) are estimated using the full covariance matrix. The cell esds are taken into account individually in the estimation of esds in distances, angles and torsion angles; correlations between esds in cell parameters are only used when they are defined by crystal symmetry. An approximate (isotropic) treatment of cell esds is used for estimating esds involving l.s. planes.
Refinement. Refinement of F^2^ against ALL reflections. The weighted R-factor wR and goodness of fit S are based on F^2^, conventional R-factors R are based on F, with F set to zero for negative F^2^. The threshold expression of F^2^ > 2sigma(F^2^) is used only for calculating R-factors(gt) etc. and is not relevant to the choice of reflections for refinement. R-factors based on F^2^ are statistically about twice as large as those based on F, and R- factors based on ALL data will be even larger.

### Fractional atomic coordinates and isotropic or equivalent isotropic displacement parameters (Å^2^)

**Table:**

	*x*	*y*	*z*	*U*_iso_*/*U*_eq_	
O1	0.27706 (14)	0.44090 (19)	0.44732 (12)	0.0641 (5)	
H1	0.2994	0.5168	0.4665	0.096*	
O2	0.11097 (14)	0.54658 (17)	0.41482 (10)	0.0518 (5)	
O3	0.18983 (14)	0.55768 (18)	0.23240 (12)	0.0636 (5)	
O4	0.34420 (14)	0.42876 (17)	0.26978 (12)	0.0589 (5)	
H4	0.3756	0.5037	0.2794	0.088*	
O5	−0.04992 (17)	0.3310 (2)	0.45621 (11)	0.0667 (5)	
H5	−0.0883	0.3681	0.4832	0.100*	
O6	−0.15670 (16)	0.4586 (2)	0.35120 (11)	0.0692 (6)	
O7	−0.05944 (14)	0.47589 (18)	0.07724 (9)	0.0553 (5)	
O8	0.10445 (14)	0.3927 (2)	0.06854 (10)	0.0578 (5)	
H8	0.0852	0.4324	0.0229	0.087*	
C11	0.0262 (2)	0.4068 (2)	0.10675 (14)	0.0448 (6)	
C12	0.04494 (19)	0.3292 (2)	0.18872 (13)	0.0434 (6)	
H12	0.0147	0.2365	0.1738	0.052*	
C13	−0.02428 (18)	0.3930 (2)	0.24293 (13)	0.0439 (6)	
H13A	−0.0030	0.4887	0.2537	0.053*	
H13B	−0.1037	0.3900	0.2120	0.053*	
C14	−0.00665 (19)	0.3186 (2)	0.32665 (14)	0.0447 (6)	
H14	−0.0332	0.2241	0.3130	0.054*	
C15	−0.0792 (2)	0.3797 (3)	0.37755 (15)	0.0510 (6)	
C16	0.11869 (19)	0.3086 (2)	0.37600 (14)	0.0439 (6)	
H16	0.1234	0.2463	0.4236	0.053*	
C17	0.1676 (2)	0.4448 (3)	0.41336 (13)	0.0448 (6)	
C18	0.1851 (2)	0.2407 (2)	0.32092 (14)	0.0462 (6)	
H18A	0.1610	0.1457	0.3110	0.055*	
H18B	0.2646	0.2405	0.3517	0.055*	
C19	0.17057 (19)	0.3116 (2)	0.23536 (14)	0.0426 (6)	
H19	0.2032	0.2509	0.2011	0.051*	
C20	0.23417 (19)	0.4466 (2)	0.24532 (13)	0.0434 (6)	
N1	0.64426 (18)	1.3190 (2)	0.48929 (14)	0.0598 (6)	
N2	0.45114 (19)	0.6692 (2)	0.31157 (14)	0.0628 (6)	
C1	0.3891 (2)	0.7823 (3)	0.2905 (2)	0.0752 (9)	
H1A	0.3180	0.7743	0.2521	0.090*	
C2	0.5531 (2)	0.6854 (3)	0.36420 (18)	0.0708 (8)	
H2	0.5992	0.6083	0.3784	0.085*	
C3	0.5951 (2)	0.8100 (3)	0.39928 (17)	0.0642 (8)	
H3	0.6675	0.8154	0.4360	0.077*	
C4	0.4243 (2)	0.9105 (3)	0.32249 (19)	0.0713 (8)	
H4A	0.3774	0.9863	0.3056	0.086*	
C5	0.5293 (2)	0.9264 (3)	0.37970 (15)	0.0502 (6)	
C6	0.5699 (2)	1.0628 (3)	0.41730 (15)	0.0510 (6)	
C7	0.6679 (2)	1.0774 (3)	0.4825 (2)	0.0817 (10)	
H7	0.7114	1.0005	0.5039	0.098*	
C8	0.7015 (2)	1.2050 (3)	0.5157 (2)	0.0809 (10)	
H8A	0.7682	1.2116	0.5592	0.097*	
C9	0.5115 (2)	1.1812 (3)	0.39072 (19)	0.0710 (8)	
H9	0.4452	1.1782	0.3466	0.085*	
C10	0.5494 (2)	1.3050 (3)	0.42825 (19)	0.0770 (9)	
H10	0.5059	1.3829	0.4096	0.092*	

### Atomic displacement parameters (Å^2^)

**Table:**

	*U*^11^	*U*^22^	*U*^33^	*U*^12^	*U*^13^	*U*^23^
O1	0.0506 (10)	0.0539 (12)	0.0736 (12)	0.0025 (9)	−0.0055 (9)	−0.0150 (9)
O2	0.0551 (10)	0.0423 (10)	0.0511 (10)	0.0043 (8)	0.0035 (8)	−0.0037 (7)
O3	0.0496 (10)	0.0378 (11)	0.0928 (14)	−0.0003 (8)	0.0027 (9)	0.0034 (9)
O4	0.0451 (10)	0.0509 (11)	0.0739 (12)	−0.0023 (8)	0.0055 (9)	−0.0018 (9)
O5	0.0850 (14)	0.0675 (13)	0.0526 (11)	0.0182 (10)	0.0275 (10)	0.0121 (9)
O6	0.0627 (12)	0.0789 (14)	0.0641 (12)	0.0200 (11)	0.0148 (9)	0.0068 (10)
O7	0.0520 (10)	0.0667 (12)	0.0430 (9)	0.0097 (9)	0.0066 (8)	0.0042 (8)
O8	0.0573 (11)	0.0720 (13)	0.0431 (10)	0.0095 (9)	0.0123 (8)	0.0055 (8)
C11	0.0442 (14)	0.0421 (14)	0.0432 (13)	−0.0045 (11)	0.0042 (11)	−0.0098 (10)
C12	0.0472 (13)	0.0361 (13)	0.0405 (12)	−0.0070 (10)	0.0019 (10)	−0.0041 (10)
C13	0.0406 (13)	0.0433 (14)	0.0429 (12)	−0.0008 (11)	0.0040 (10)	0.0006 (10)
C14	0.0496 (13)	0.0344 (13)	0.0472 (13)	−0.0021 (11)	0.0090 (11)	−0.0006 (10)
C15	0.0538 (15)	0.0488 (16)	0.0481 (14)	−0.0035 (13)	0.0106 (12)	0.0012 (12)
C16	0.0499 (14)	0.0350 (13)	0.0435 (13)	0.0012 (11)	0.0079 (10)	0.0055 (10)
C17	0.0506 (15)	0.0448 (15)	0.0341 (12)	0.0025 (12)	0.0038 (10)	0.0021 (10)
C18	0.0528 (14)	0.0309 (13)	0.0500 (13)	0.0029 (11)	0.0064 (11)	0.0011 (10)
C19	0.0460 (13)	0.0346 (13)	0.0447 (13)	0.0006 (10)	0.0085 (10)	−0.0041 (10)
C20	0.0417 (13)	0.0461 (15)	0.0377 (12)	−0.0015 (11)	0.0037 (10)	−0.0014 (10)
N1	0.0532 (13)	0.0507 (14)	0.0681 (14)	−0.0042 (11)	0.0051 (11)	−0.0076 (11)
N2	0.0562 (14)	0.0545 (15)	0.0725 (15)	−0.0120 (12)	0.0098 (12)	−0.0052 (11)
C1	0.0527 (17)	0.064 (2)	0.097 (2)	−0.0059 (15)	0.0005 (15)	−0.0144 (17)
C2	0.0712 (19)	0.0468 (17)	0.081 (2)	−0.0035 (14)	0.0001 (16)	0.0061 (14)
C3	0.0571 (16)	0.0519 (17)	0.0703 (18)	−0.0051 (13)	−0.0037 (13)	0.0025 (13)
C4	0.0489 (16)	0.0555 (18)	0.096 (2)	−0.0009 (13)	−0.0007 (15)	−0.0136 (15)
C5	0.0472 (14)	0.0479 (16)	0.0551 (14)	−0.0075 (12)	0.0140 (11)	−0.0017 (11)
C6	0.0438 (14)	0.0524 (16)	0.0554 (15)	−0.0050 (12)	0.0116 (11)	−0.0009 (12)
C7	0.0556 (17)	0.0535 (19)	0.113 (3)	0.0029 (14)	−0.0137 (17)	−0.0099 (17)
C8	0.0566 (18)	0.063 (2)	0.100 (2)	−0.0029 (16)	−0.0161 (16)	−0.0139 (17)
C9	0.0581 (17)	0.0552 (18)	0.0795 (19)	−0.0018 (14)	−0.0135 (14)	−0.0039 (14)
C10	0.0664 (19)	0.0549 (19)	0.090 (2)	0.0023 (15)	−0.0099 (16)	−0.0040 (15)

### Geometric parameters (Å, °)

**Table:**

O1—C17	1.310 (3)	C18—H18A	0.9700
O1—H1	0.8200	C18—H18B	0.9700
O2—C17	1.215 (3)	C19—C20	1.515 (3)
O3—C20	1.202 (3)	C19—H19	0.9800
O4—C20	1.315 (3)	N1—C10	1.319 (3)
O4—H4	0.8200	N1—C8	1.321 (3)
O5—C15	1.332 (3)	N2—C2	1.321 (3)
O5—H5	0.8200	N2—C1	1.329 (4)
O6—C15	1.207 (3)	C1—C4	1.375 (4)
O7—C11	1.231 (3)	C1—H1A	0.9300
O8—C11	1.301 (3)	C2—C3	1.379 (4)
O8—H8	0.8200	C2—H2	0.9300
C11—C12	1.508 (3)	C3—C5	1.378 (4)
C12—C13	1.532 (3)	C3—H3	0.9300
C12—C19	1.534 (3)	C4—C5	1.380 (3)
C12—H12	0.9800	C4—H4A	0.9300
C13—C14	1.520 (3)	C5—C6	1.490 (3)
C13—H13A	0.9700	C6—C9	1.364 (4)
C13—H13B	0.9700	C6—C7	1.381 (4)
C14—C15	1.512 (3)	C7—C8	1.372 (4)
C14—C16	1.535 (3)	C7—H7	0.9300
C14—H14	0.9800	C8—H8A	0.9300
C16—C17	1.513 (3)	C9—C10	1.374 (4)
C16—C18	1.534 (3)	C9—H9	0.9300
C16—H16	0.9800	C10—H10	0.9300
C18—C19	1.535 (3)		
			
C17—O1—H1	109.5	H18A—C18—H18B	107.6
C20—O4—H4	109.5	C20—C19—C12	112.18 (19)
C15—O5—H5	109.5	C20—C19—C18	111.54 (18)
C11—O8—H8	109.5	C12—C19—C18	110.52 (19)
O7—C11—O8	122.5 (2)	C20—C19—H19	107.5
O7—C11—C12	121.7 (2)	C12—C19—H19	107.5
O8—C11—C12	115.7 (2)	C18—C19—H19	107.5
C11—C12—C13	110.54 (19)	O3—C20—O4	123.5 (2)
C11—C12—C19	112.56 (19)	O3—C20—C19	124.3 (2)
C13—C12—C19	113.81 (18)	O4—C20—C19	112.2 (2)
C11—C12—H12	106.5	C10—N1—C8	116.2 (2)
C13—C12—H12	106.5	C2—N2—C1	116.4 (2)
C19—C12—H12	106.5	N2—C1—C4	123.6 (3)
C14—C13—C12	112.17 (19)	N2—C1—H1A	118.2
C14—C13—H13A	109.2	C4—C1—H1A	118.2
C12—C13—H13A	109.2	N2—C2—C3	123.8 (3)
C14—C13—H13B	109.2	N2—C2—H2	118.1
C12—C13—H13B	109.2	C3—C2—H2	118.1
H13A—C13—H13B	107.9	C5—C3—C2	119.8 (2)
C15—C14—C13	111.2 (2)	C5—C3—H3	120.1
C15—C14—C16	113.39 (19)	C2—C3—H3	120.1
C13—C14—C16	112.16 (19)	C1—C4—C5	119.9 (3)
C15—C14—H14	106.5	C1—C4—H4A	120.1
C13—C14—H14	106.5	C5—C4—H4A	120.1
C16—C14—H14	106.5	C3—C5—C4	116.5 (2)
O6—C15—O5	123.2 (2)	C3—C5—C6	121.8 (2)
O6—C15—C14	125.7 (2)	C4—C5—C6	121.7 (2)
O5—C15—C14	111.1 (2)	C9—C6—C7	115.6 (2)
C17—C16—C18	113.58 (19)	C9—C6—C5	122.2 (2)
C17—C16—C14	112.76 (19)	C7—C6—C5	122.3 (2)
C18—C16—C14	109.87 (18)	C8—C7—C6	120.2 (3)
C17—C16—H16	106.7	C8—C7—H7	119.9
C18—C16—H16	106.7	C6—C7—H7	119.9
C14—C16—H16	106.7	N1—C8—C7	123.7 (3)
O2—C17—O1	123.0 (2)	N1—C8—H8A	118.2
O2—C17—C16	123.7 (2)	C7—C8—H8A	118.2
O1—C17—C16	113.2 (2)	C6—C9—C10	120.9 (2)
C16—C18—C19	114.08 (18)	C6—C9—H9	119.6
C16—C18—H18A	108.7	C10—C9—H9	119.6
C19—C18—H18A	108.7	N1—C10—C9	123.4 (3)
C16—C18—H18B	108.7	N1—C10—H10	118.3
C19—C18—H18B	108.7	C9—C10—H10	118.3

### Hydrogen-bond geometry (Å, °)

**Table:**

*D*—H···*A*	*D*—H	H···*A*	*D*···*A*	*D*—H···*A*
O1—H1···N1^i^	0.82	1.81	2.630 (3)	177
O4—H4···N2	0.82	1.86	2.678 (3)	175
O5—H5···O2^ii^	0.82	1.96	2.723 (2)	153
O8—H8···O7^iii^	0.82	1.82	2.641 (2)	174

Symmetry codes: (i) −*x*+1, −*y*+2, −*z*+1; (ii) −*x*, −*y*+1, −*z*+1; (iii) −*x*, −*y*+1, −*z*.
